# The Universities and Medicine

**Published:** 1915-10-23

**Authors:** 


### THE UNIVERSITIES AND MEDICINE.

The times have gone by when it was regarded as an undignified thing for a University to concern itself with the teaching of subjects having a utilitarian value. In odd nooks and corners the creed still lingers, but it makes little open boast and receives no official countenance. Even in quarters where the newer developments are deplored the necessity for them is recognised. Hence without challenge the training of doctors now takes its stand as a legitimate University function, and a medical faculty is so well established that it is regarded as quite in the ordinary course of things. In one sense this development is not a new one. There have always been Universities where the teaching of medicine was actively pursued, and others have professed the name if not the substance. It is the change in these latter from a merely nominal organisation to an effective one which is the conspicuous event. Doubtless the establishment of many new Universities in the busy centres of practical life has done much in England to produce this change of opinion, but this has not been the sole influence at work. Something is due to the conviction that it is less the subject than the manner of teaching it which constitutes the true academic quality. Another influence is, we think, to be found in the tendency, perhaps an inevitable one, to extreme specialisation in the training provided in Technical Schools and Colleges. From this, perhaps in a rather vague fashion, has arisen the feeling that some broader and more general basis is necessary to a sound education, and this demand has had a stimulating effect upon University conceptions. At the present moment, under the pressure of immediate circumstances, some sections of the public seem to think that the Universities ought to be the very homes of utilitarian knowledge, and they are being blamed for their failure to give birth to aniline dyes and high explosives. In time a more correct perspective will be restored, though the Universities may usefully take note of the expectation that they are expected to minister to the national life and not to dwell on some lofty height remote from the common lot. If it has been an advantage to medicine to stand in its due place in the academic world, there has been some reciprocal benefit, for the association has helped to keep this world in touch with the larger school of life.

The immediate influence of the Universities on medicine falls, of course, on the medical student. In his earlier scientific studies he is made conscious of the broad conceptions which these present, and thus has an opportunity of obtaining from them their full educational value. The bearing of such studies on medical opinion and practice is necessarily and properly emphasised, but something more is added. There is thus provided a wider outlook than in organisations where the sense of impending practice dominates everything and a hurried movement towards what are called the practical subjects is inevitably encouraged. A University training, in short, assuming it is worthy of the name, makes each part of the curriculum contribute something to the mental discipline and training of the student; its aim is something more than a mere technical equipment which may or may not have a field of application in medical or surgical practice. Hence, if he has used his opportunities, the medical graduate has acquired a mental attitude which will influence not only his work, but his judgment of things and his outlook on life. Whatever arts, or crafts, or dexterities he may have acquired he has been provided with what in the end is even more important—namely, the possibility of development. It would be to write without practical experience of men and manners to say that the educational result just defined is never found apart from a University training, just as it would be to deny evidence to propose that every University graduate reaches the ideal. Men of force and fibre grow whatever the environment, and, on the other hand, there is the person who, though brayed even in the University "mortar . . . yet will not his foolishness depart from him."

Allowing both of these possibilities, it still remains true that for the majority a University training provides an educational influence which saves from narrowness and hardness of spirit, and without which life to them would have had a less dignified ambition and a less expansive horizon. We are far from asserting that these highly desirable qualities come solely from contact with teaching organised on academic lines and conducted by men of academic rank. Not always is the man equal to his rank, and too much must not be expected even from professors. Fortunately, University influences include other educational forces, and not the least of these is the sustained association with minds which have other qualities and other ambitions than our own. It is a real gain to medicine that her young disciples in University life are in contact with contemporaries who cultivate arts, or divinity, or law, or science, or vaguely things in general. Out of such contact grow not only helpful friendships but large views and the stimulating influences of debate. The full force of this tide is doubtless in the residential Universities, but it has high force elsewhere, for youth will find its fellows even though all are not under a common roof nor guests at a common table.

